# Nervous Sore Throat

**Published:** 1874-07-01

**Authors:** 

**Affiliations:** Deutsch Klinik


### Gleanings from Our Exchanges.

### NERVOUS SORE THROAT.

ABSTRACT OF AN ARTICLE BY DR. KLEMM, IN THE "DEUTSCH KLINIK."

*From the Chicago Journal of Nervous and Mental Disease, April* 1874.

A VERY large number of sufferers from so-called sore throat, complain of a constant painful sensation without showing any other than the most trifling morbid alterations in the region affected. Such patients, who cannot on other considerations be accounted as nervous cases, often occupy, more than any others, the time of the physician, and frequently have to be accounted as absolutely incurable.

"In such cases, even the closest examination affords no satisfactory explanation why the abnormal sensation should appear in the perfectly normal mucous membrane. The search for an anatomical cause has, in this matter, afforded us no satisfactory explanation; on the contrary, it frequently occurs that altogether unimportant and secondary alterations are mistaken for the *corpus delicti*, and, in consequence, a false system of treatment is decided upon, and the stronger caustic agents are, especially, misemployed. Quite often, very trifling alterations of the mucous membrane of the throat are taken to be the source of the numerous and often very different sensations experienced in the throat and larynx (in drinking, speaking, or in rest, etc.): such as the well-known small, isolated, hypertrophic, warts or band-like excrescences, or even the almost constant thickening of the lining membrane with increased secretion of mucus in old persons, which, if it occurs together with any nervous complaint, is usually believed to be the cause: so that the whole is considered as a chronic throat catarrh. In very many cases these alterations are present without producing any uncomfortable symptoms of pricking, pressure, constriction, choking, etc., and they are very often wanting when these troubles are present in a very pronounced and rebellious form.

"In many such cases of sore throat we find, to be sure, some swelling of the mucous membrane, the submucous cellular tissue, and the glands, especially in the throat; and in these cases the pain is relieved by caustic applications. But the author excepts these cases of true chronic pharyngitis, and confines himself more exclusively to those in which the pharyngitis is either extremely slight or altogether lacking. In like manner, many cases of pulmonary disease, with normal mucous membrane, would be erroneously diagnosed as chronic pharyngitis, and treated with caustics, without profit, if we take into account only the pain in the throat and the feeling of pressure, etc.

"The results of treatment show, moreover, that the caustics have either a very slight effect, or none at all; and that only one thing proves beneficial, that is, change of air, and the milk-cure, with the employment of some mineral waters, which are here often of great service.

"The essential symptom of these various forms of hyperæsthesia of the pharynx is the difficulty experienced in swallowing, or in speech. Swallowing is always accompanied with an abnormal sensation; the patient complains of pressure, pricking, or sensation of constriction, or the feeling of some foreign body, sometimes as if a hair was lodged in the throat. The painful sensation either is felt on both sides, or it may be confined to a single point, accurately designated by the sufferer. Sometimes it is constantly present; but it is generally periodically milder, often lacking entirely, and then again severe. In the evening it is always more severe than in the forenoon, and, in many cases, returns daily, in the afternoon. Emotions of all kinds have a bad influence, especially upon those who have lost relatives from consumption, or who are particularly fearful and nervous. Many of the patients complain of a dryness of the throat, without any such appearance of the mucous membrane, an especially troublesome and constant symptom; or they affirm in the most confident manner, that there must be a foreign body lodged there, causing them to attempt to swallow, or hawk, and cough, while really no collection of thick mucus, as in actual pharyngitis, is really present. Speech is affected in sympathy; it is not hoarse, but almost inaudible, and the patients complain that it soon fatigues them and causes pain. Finally, we have the *globus hystericus*, but this is met with much more rarely than the other symptoms. Sometimes again the painful sensation extends to the ear, and hearing is affected.

"The individuals who are especially liable to this nervous affection are by no means always of a nervous or hypochondriacal disposition; it attacks frequently the female sex, and not merely hysterical or irritable women, and those in the higher walks of life, but among others, strong and healthy women, and particularly those of the lower classes, who have nothing but their throats to complain of. This hyperæsthesia is rather common among men; and, according to the author's observation, it affects the cultured more than the working class, and is not at all rare among those who are in the custom of public speaking or singing, or who have often suffered from catarrh. In both sexes he found the fear of consumption, which had caused the death of a cousin, or a brother, etc., to be an indubitable cause of the affection; and frequently a recent loss of this kind throws the patient into great agitation and establishes the disease.

"Very often the psychic origin may be detected when there also exists an ordinary nasal or bronchial catarrh, without any participation of the membranes of the throat; and in this case, also, fear is the principal cause of the disease. A third cause is yet to be mentioned, the persistent excitability often remaining after an acute pharyngitis or laryngitis, similar to the lasting irritability of the tonsils, without hypertrophy, after an acute amygdalitis. This is the case not only after acute but also after subacute inflammations of these parts, which are readily re-incited, and which leave the throat for a considerable period in quite an irritable condition. Finally, we may enumerate among its exciting causes, external irritation from wind, dust, indulgence in stimulants (even coffee), which very easily produce hyperæsthesia in sensitive subjects, without any corresponding alteration in the mucous membrane. It is sometimes very difficult to decide, in cases where there are slight alterations, whether the actual very insignificant and habitual abnormal appearances are really the cause of the trouble or not; and only by prolonged observation can a correct opinion be given. Whether the affection is ever hereditary, is doubtful; but the patients will sometimes so assert.

"The participation of the vocal organs is specially noticeable in this form of hyperæsthesia; the voice is either inaudible or harsh, although nothing abnormal can be detected in the larynx; the patients unintentionally aggravate the symptoms, either because fear and imagination co-operate with the disease, or because the activity of the motor fibres is diminished. The inconstancy of the phenomena, the rapid onset of the disease after emotional disturbances, and its quick departure, prove that its cause is not a catarrhal trouble, but a purely nervous affection. Another peculiarity is in the fact that such invalids feel free from their difficulty in the open air, while they suffer in-doors; and correspondingly, we find this purely nervous hyperæsthesia much more rarely among dwellers in the country than among townspeople, although they are often enough the subjects of chronic pharyngitis, and are made worse by raw air or draughts.

"The cure of this affection is one of the most difficult tasks of the physician; the patients often engage his attention for years without obtaining relief; and even if a cure seems to be obtained, they again readily relapse. Here, also, appears the difference between the nervous and the catarrhal form: the latter is altogether more yielding to local remedies, while in the other case they very often are of no use, or are merely of transient effect, and their employment seems to be very little indicated, as they only afford a momentary alleviation. Frequently they are even injurious, since by their use the hyperæsthesia is increased.

"The inexperienced physician is readily inclined to consider the disease altogether imaginary; but this is not the fact; it really exists, and is much more important than many others with visible alterations for a cause, and which are suitable for treatment with nitrate of silver in substance.

"The treatment is based on very slight foundations. If material alterations of the mucous membrane present themselves, it is always justifiable to apply local applications; and if it is desired to remove red fleshy excrescences, the caustics in substance are preferable to weak solutions. But if these are lacking, the action of weak solutions (0.3 to 15 or 30 water), is indicated; or we may pencil the parts with chloroform and glycerine, which is sometimes of service. If there is no chronic catarrh, we may try electricity, which sometimes causes a rapid improvement in rebellious cases; but it is needful that one electrode, armed with a sponge, be placed directly upon the mucous surface, while the other is applied at different points of the external surface. If nitrate of silver is used, after other treatment has failed, strong solutions should at all events be avoided, and the weak solution should be applied over the whole surface of the pharynx, and especially over that portion below and behind the tongue. Dr. Klemm has used, instead of glycerine, a solution of morphia with mucilage, and has found that this means has a better effect than the astringents.

"The most effectual treatment in this, as in other disorders of nervous activity, is, according to the author, a change of air; and mountain air is, by all means, the most beneficial; after it comes the sea air. Among mountain localities, those must be chosen which are moderately high and well protected; and those elevated situations which are recommended for lung complaints are unsuitable, as the raw, dry atmosphere only aggravates the evil. If it is not possible for the patient to visit the mountains or the sea-shore he should be sent into the country, and treated by the milk-cure and mineral waters. The atmospheric change is still the principal point, and the good results attainable by residence at watering and bathing places are doubtless due not to the high-priced mineral waters, but to the favorable situation and climate. The fact that in the various localities the most different agents are employed with equal results, shows plainly that the cure does not depend upon these, but on the effect of the atmosphere on the nerves and mucous membranes."

